# Supplementary material for: Biochemical Characterization and Functional Analysis of Glucose Regulated Protein 78 from the Silkworm Bombyx mori
Source: Int J Mol Sci. 2023 Feb 16;24(4):3964. doi: 10.3390/ijms24043964 (PMC9961775; doi:10.3390/ijms24043964)
Supplement: Supplementary file 1 [file ijms-24-03964-s001.zip › ijms-2180888-supplementary.pdf]

Biochemical Characterization and Functional Analysis of Glucose Regulated Protein 78 from the silkworm, *Bombyx mori*

Yao Xiao [1, 2 †], Lujie Ren[1, 2 †], Yanan Wang [1, 2], Huanhuan Wen [1, 2], Yongqiang Ji [1, 2], Chenshou Li [3], Yangqing Yi [1, 2], Caiying Jiang [1, 2], Qing Sheng [1, 2], Zuoming Nie [1, 2], Qixiang Lu [1, 2], Zhengying You [1, 2] *

1. College of Life Sciences and Medicine, Zhejiang Sci-Tech University, Hangzhou 310018, China

2. Zhejiang Provincial Key Laboratory of Silkworm Bioreactor and Biomedicine, College of Life Sciences and Medicine, Zhejiang Sci-Tech University, Hangzhou 310018, China

3. Engineering Research Center for Eco-Dyeing and Finishing of Textiles, Ministry of Education, Zhejiang Sci-Tech University, Hangzhou 310018, China.

* Correspondence: zyyou@zstu.edu.cn

[†] These authors contributed equally to this work.

This file includes:

Supporting Information

Figures S1-S4

Supplementary materials:

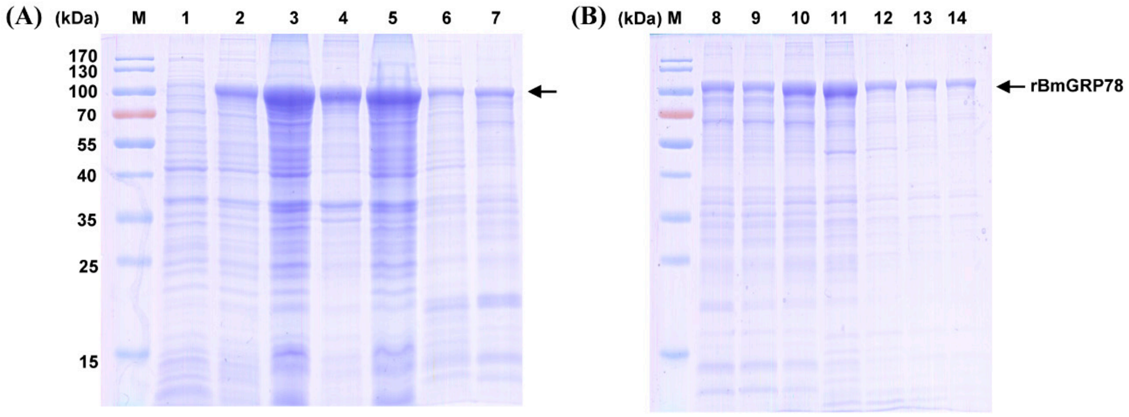

Figure S1. SDS-PAGE analysis for the purification of rBmGRP78 protein. (A) shows SDS-PAGE analysis of rBmGRP78 expression and purification in *E. coli* BL21 (DE3) induced by IPTG. Lane M: Protein Molecular Weight Marker; Lane 1: pET-32a(+)-*rBmGRP78* in BL21, no IPTG treatment; Lane 2: pET-32a(+)-*rBmGRP78* in BL21, IPTG treatment; Lane3: Supernatant of pET-32a(+)-*rBmGPR78* in BL21, IPTG treatment; Lane4: Precipitation of pET-32a(+)-*rBmGRP78* in BL21, IPTG treatment; Lane5-7: Imidazole elution 25mM-1, 25mM-2, 50mM-1; **(B)** Lane 8-14: Imidazole elution 50mM-2, 50mM-3, 100mM, 300mM, 500mM-1, 500mM-2, 500mM-3.

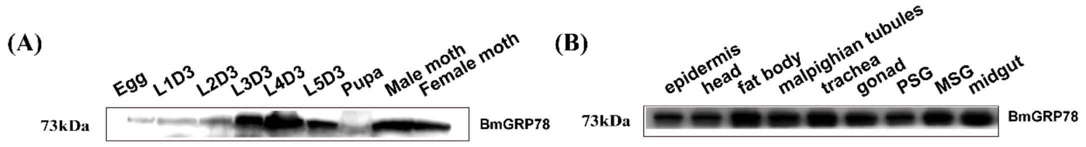

Figure S2. Western blotting analysis for expression patterns of BmGRP78 in different stages and tissues of *Bombyx mori.* Identification of the BmGRP78 translation level in different developmental stages **(A)** and tissues of L5D3 larvae **(B)** by Western blotting.

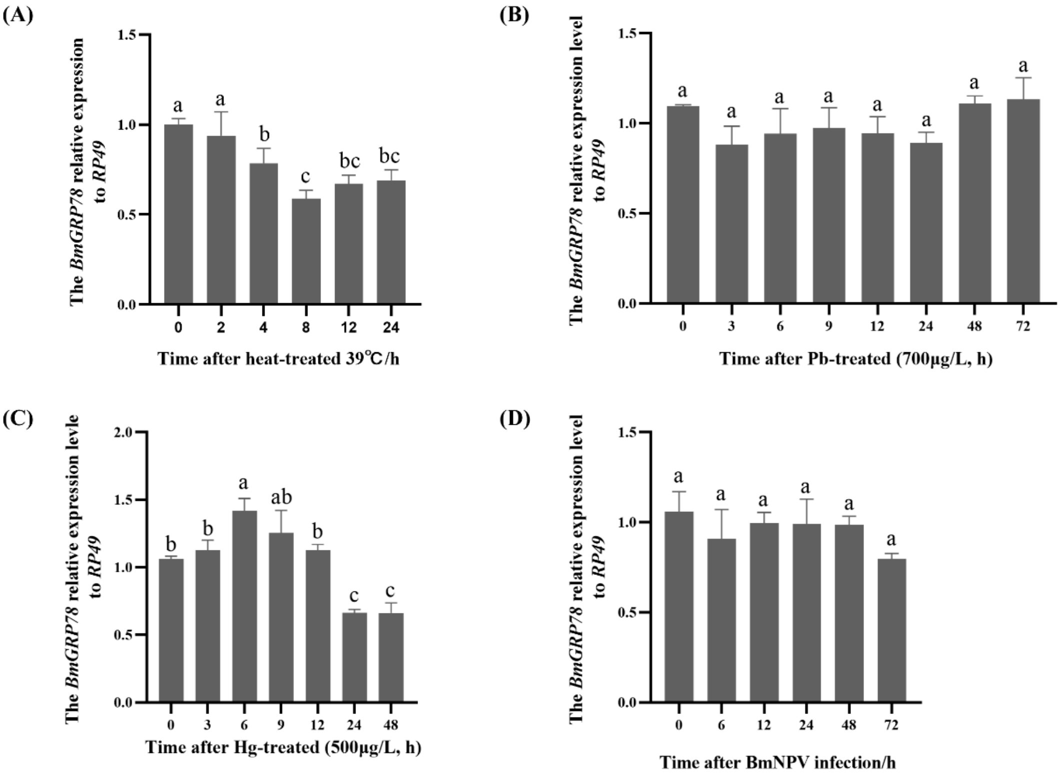

Figure S3. The transcript level of *BmGRP78* in response to different stress-induced conditions. (A) Heat-treated, **(B)** Pb-treated, **(C)** Hg-treated, **(D)** BmNPV infection. Bars represent mean ± S.E.(n=3). Bars with different letters are significantly different (one-way ANOVA followed by Tukey's test, $p < 0.05$).

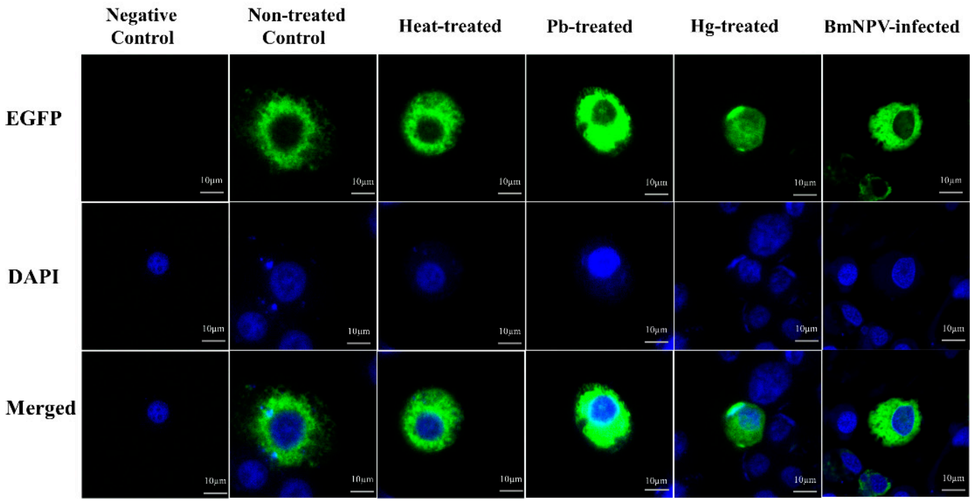

Figure S4. Subcellular localization of BmGRP78 under different stress-induced conditions in BmN4 cells using EGFP-tagged clone. The pIEx-BmGRP78-EGFP construct was transiently expressed in BmN4 cells. The cell nuclei were stained with DAPI (blue). The fluorescent signal was imaged by confocal microscopy. EGFP green fluorescence, DAPI blue fluorescence, and merged images are shown.
